# A Rare Case of Metastatic Pulmonary Sarcomatoid Carcinoma

**DOI:** 10.7759/cureus.89143

**Published:** 2025-07-31

**Authors:** Robert E Carter, Krista L Denning, Lynne J Goebel

**Affiliations:** 1 Internal Medicine, Joan C. Edwards School of Medicine at Marshall University, Huntington, USA; 2 Pathology, Joan C. Edwards School of Medicine at Marshall University, Huntington, USA; 3 Internal Medicine and Geriatrics, Joan C. Edwards School of Medicine at Marshall University, Huntington, USA

**Keywords:** atypical chest pain, metastatic non-small cell lung cancer, programmed cell death ligand 1, pulmonary sarcomatoid carcinoma (psc), smoking tobacco

## Abstract

Pulmonary sarcomatoid carcinoma (PSC) is a rare, aggressive cancer with early metastasis and poor prognosis. Chest X-ray and computed tomography (CT) are common modalities used to detect malignancy, and the diagnosis is confirmed with tissue biopsy. There are currently no guidelines for therapy when treating this condition as it often displays resistance to typical first-line chemotherapy agents. We present a case of PSC with brain metastasis and our approach to management to remind clinicians of this rare condition and the limited choices of treatment.

## Introduction

Pulmonary sarcomatoid carcinoma (PSC) is an uncommon form of non-small cell lung carcinoma (NSCLC) that represents 0.3% to 3% of all malignant lung tumors and consists of epithelial and mesenchymal characteristics [1]. It can further be classified into pleomorphic carcinoma, carcinosarcoma, spindle cell carcinoma, giant cell carcinoma and pulmonary blastoma [2]. PSC is a rapidly growing form of lung cancer that has both sarcoma and carcinoma characteristics [2]. This type of cancer has a poor prognosis, with a median overall survival (OS) of 256 days with chemotherapy; this was not significantly different than with no therapy at all in a study of 25 patients at one medical center [3].

The purpose of this case report is to describe the diagnosis and management of a patient with PSC, with the aim of highlighting the challenges faced in the management of this disease. We describe the clinical presentation, imaging findings, histological examination, and treatment outcome of a patient with PSC. The information presented in this report contributes to the growing body of knowledge on this rare and aggressive form of lung cancer and may inform areas for future research in its treatment.

## Case presentation

**Figure 1:**
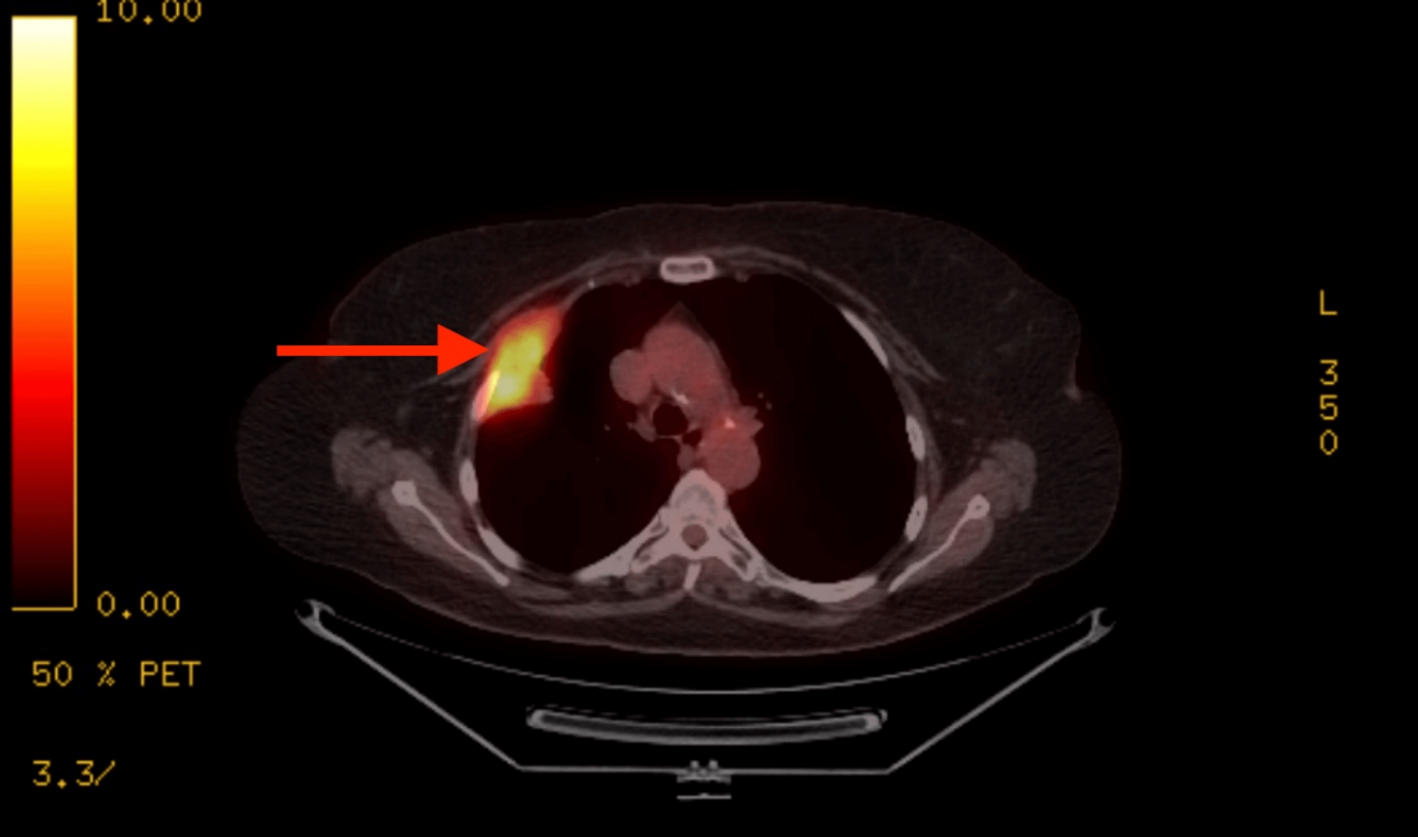
Positron emission tomography (PET) CT shows a large mass (arrow) within the right upper lobe extending to the chest wall and involving the second and third right ribs (Standardized Uptake Value 8.8 g/ml)

A female patient, aged 78, sought medical attention upon moving from Tennessee. She had a history of hyperlipidemia, varicose veins, allergic rhinitis, left-side deafness, and macular degeneration. The patient had a 45-pack-year smoking history but quit smoking 30 years ago and consumed three to four beers per week. Initially, she experienced intermittent burning chest pain, treated with antacids in the urgent care clinic. However, due to increasing frequency and severity of the chest pain over the next two months, a chest X-ray was performed, revealing a lung mass. A chest computed tomography (CT) showed a 7 cm x 3.4 cm mass in the right upper lobe infiltrating the nearby chest wall. A whole-body positron emission tomography (PET) CT scan confirmed a hyper-metabolic mass in the right upper lobe, small nodules in the lungs, and increased uptake in various regions of the body, including the cecum (Figure 1). 

**Figure 2:**
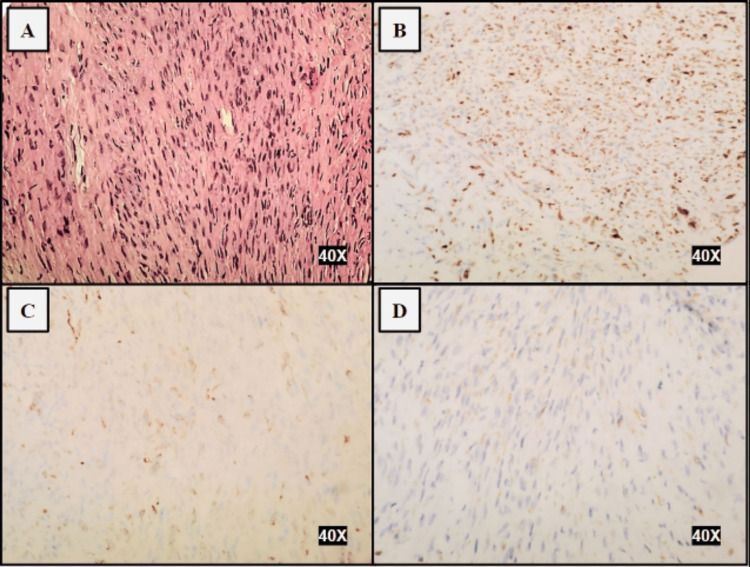
40X magnification of A. Pulmonary SC with spindle cell features (Hematoxylin and Eosin), B. CAM 5.2, C. Cytokeratin OSCAR, and D. Cytokeratin AE1/AE3 all showing focal positive staining.

The patient had no previous history of cancer. A CT-guided biopsy of the right-sided chest wall mass produced atypical spindle cells on microscopic evaluation. The spindle cells showed focal positive staining for cytokeratin CAM 5.2, cytokeratin OSCAR, and cytokeratin AE1/AE3 (Figure 2). 

The tumor cells were negative for p40, S-100, desmin, smooth muscle actin, CD34 and TTF-1. The morphology and staining pattern were consistent with PSC. Programmed death-ligand 1 (PD-L1) Tumor Proportion Score was 0. Biomarker findings revealed a microsatellite status of MS-Stable and a tumor mutational burden (TMB) of 8 Muts, both of these with no associated therapies or clinical trials. There were no other reportable alterations with diagnostic claims on next-generation sequencing.

**Figure 3:**
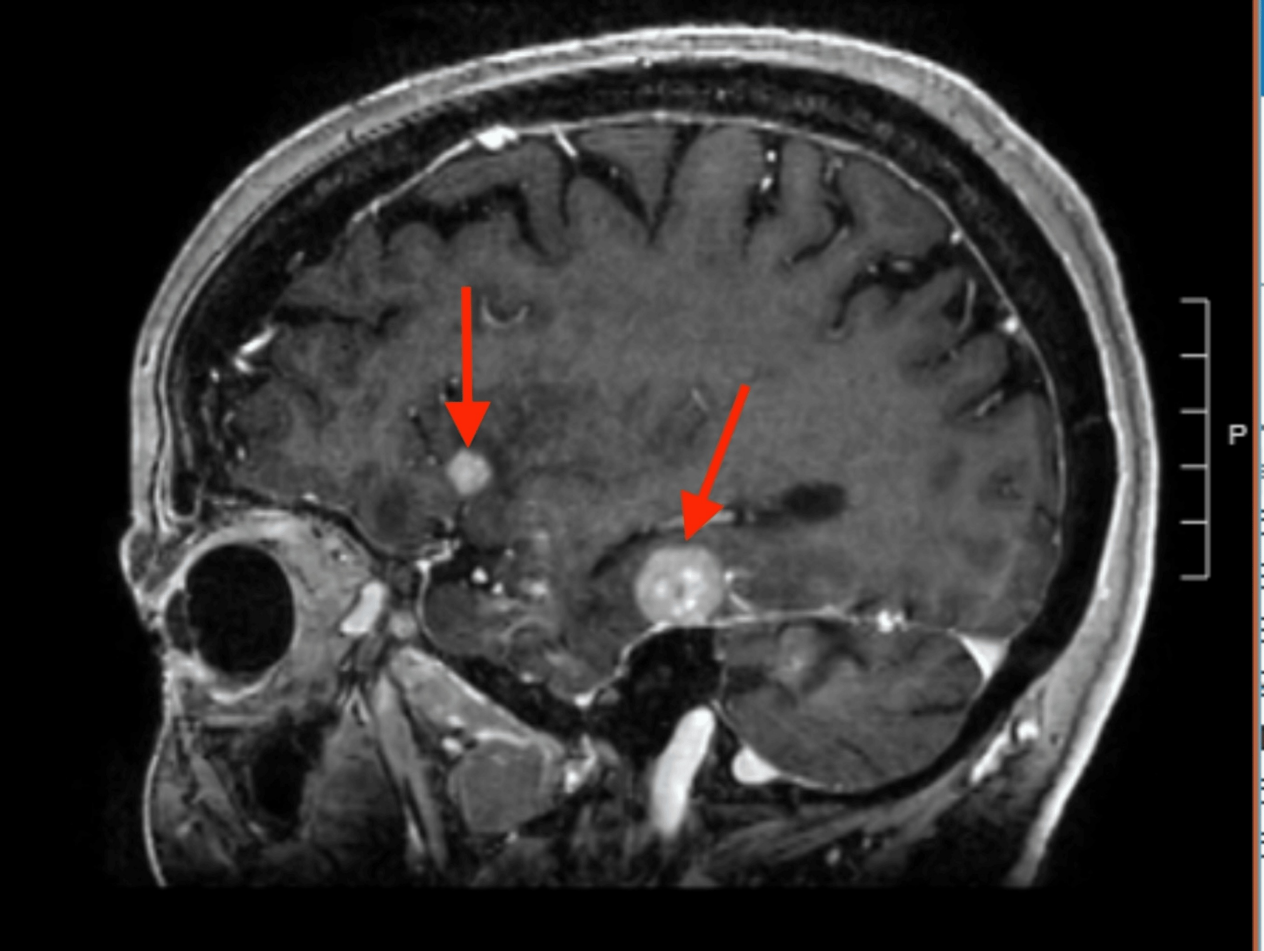
MRI Brain with contrast shows multiple metastatic lesions (arrows)

One week after her biopsy, the patient presented to the hospital with complaints of right-sided sensory abnormalities. A brain magnetic resonance image (MRI) showed multiple enhancing foci bilaterally, including the posterior fossa, consistent with metastases and associated with vasogenic edema (Figure 3). 

The patient received dexamethasone and underwent hippocampus-sparing whole-brain radiotherapy. After completing the radiation therapy, the patient reported the acute onset of severe generalized weakness and left-sided pleuritic chest pain. A CT angiogram revealed no blood clots, but showed a left lung pneumonia, leading to a prescription for antibiotics. Treatment of her cancer was delayed until she finished her antibiotics. However, the patient still reported various symptoms such as double vision, and numbness in her right arm and hand. She started palliative paclitaxel protein-bound particle/carboplatin/atezolizumab treatment.

After completing her first chemotherapy cycle, the patient experienced extreme fatigue, somnolence, decreased activity and appetite, and had a fall at home. As a result, the next cycle was postponed. During a telehealth visit, the patient opted for single-agent immunotherapy with nivolumab but developed redness on her left cheek after treatment that was felt to be an allergic reaction. Her tumor was also negative for PD-L1, giving her a lower chance of response to immunotherapy. She requested a hospice referral for palliative care. She was able to spend a couple of weeks with her daughter. Unfortunately, the patient's lifespan was only around six months from the time of initial presentation until her death.

## Discussion

Our patient had PSC, an uncommon and rapidly advancing form of NSCLC that presents significant diagnostic and therapeutic challenges. Primary PSC is an unusual malignant neoplasm with histology having both carcinoma and sarcoma-like characteristics [2]. Similar biphasic tumors have been found in the urinary tract [4], esophagus [5], skin [6], and breast [7]. From the Surveillance, Epidemiology and End Results (SEER) database of about 5000 patients with PSC, they are frequently large tumors at presentation with a median size of 5 cm [8]. Our patient’s tumor was very large at 7 cm on presentation. PSC occurs more often in older adults, with a median age of 68 years, and in the SEER database, 60% were male [8]. Symptoms can include chest pain, as in our patient, or cough and hemoptysis [9]. Smoking is also more common in PSC and along with weight loss usually alerts the astute clinician to the possibility of cancer.

The proposed process involved in PSC is epithelial-mesenchymal transition (EMT) [10]. During EMT, biochemical changes occur in an epithelial cell, including the downregulation of epithelial markers like E-cadherin and the upregulation of mesenchymal markers such as N-cadherin and vimentin, giving it mesenchymal cell properties that allow it to spread, infiltrate and resist apoptosis, which may explain the aggressiveness of PSC. Also present in PSC are more and recurring genetic mutations compared to other pulmonary neoplasms [9]. 

PSC can be classified under a spectrum of tumors that includes pleomorphic carcinoma, spindle cell carcinoma, giant cell carcinoma, carcinosarcoma, and pulmonary blastoma [2]. These tumors can be classified by light microscopy alone using both hematoxylin and eosin (H and E) staining and immunohistochemistry (IHC) [11]. Based on CT diagnosis of PSC, lesions are limited to the upper lobe and frequently peripheral [9]. There are many distinguishing factors that help differentiate between the subclasses of PSC. These include metastasis locations, histologic differences between tumors, and states of differentiation. Another two identifying features include angiotropism (cancer residing on or near the outer walls of vascular surfaces) and emperipolesis (cancer that lives within the cytoplasm of other somatic cells) for spindle cell carcinoma and giant cell carcinoma, respectively [1]. Knowledge of the histological criteria and the most common considerations in the differential diagnosis will aid in selection of appropriate IHC studies. Coupling the pathologic findings with the clinical history and distribution of the disease on imaging should help avoid misdiagnosis. Delay in diagnosis is also problematic in this disease. Our patient’s pathology was sent to an outside institution for a second opinion, which delayed the diagnosis by several days.

The treatment of PSC remains a challenge due to its ability to survive conventional therapies and its aggressive nature [9]. Surgery provides the best OS in early stages, while conventional therapies such as platinum-based chemotherapy and radiotherapy are associated with worse outcomes [9]. Other treatment options, such as epidermal growth factor receptor (EGFR)-targeted therapies, are being explored [9]. Recent advances in the understanding of the mechanisms of immune escape of tumors paved the way for new therapeutic strategies. Immune checkpoint inhibitors (ICIs) are a relatively new form of immunotherapy that are believed to have the ability to significantly improve prognosis with NSCLC [11]. For instance, programmed death receptor (PD-1)/PD-L1 interaction is recognized as a key mechanism of immune evasion in NSCLC, and ICIs such as nivolumab are used to treat tumors that express PD-L1 [12]. In a study testing nivolumab’s efficacy in 37 patients with PSC, the objective response rate was 40.5% and the disease control rate was 64.8% [12]. This suggests that targeting the PD-1/PD-L1 pathway to restore T cells’ ability to phagocytize could be an effective first-line defense against PSC, but this study is criticized for having an unusually high percentage of positive PD-L1 tumors, high TMB, and immunotherapy being second- or third-line therapy. 

Overall prognosis for PSC is poor, with a low median OS, regardless of stage or treatment. Although surgical resection shows a better prognosis in PSC, patients that undergo surgery still have a high chance of relapse. Studies show that these tumors are associated with a relatively poor prognosis whatever the stage at diagnosis, early or metastatic, and are resistant to conventional chemotherapy [8]. 

The management of PSC requires a multidisciplinary approach, with close collaboration between the primary care physician, medical oncologist, pulmonologist, surgeon, radiation oncologist, and palliative care team. The patient’s goals and expectations must be considered when making treatment decisions, as some patients may choose to pursue aggressive measures while others may opt for palliative care.

## Conclusions

In conclusion, this case report highlights the challenges and complexities associated with the diagnosis and treatment of PSC. This report serves as a reminder of the importance of considering lung cancer such as PSC in the differential diagnosis of chest wall pain in a smoker and the need for continued research and innovation in the treatment of this aggressive form of cancer. Unfortunately, patients with PSC usually present at an advanced stage. The tumor does not respond well to traditional chemotherapy and radiation, leaving patients and families distraught. The rapid course leaves little time for end-of-life planning. Despite the challenges presented by PSC, it is important to remain optimistic and continue to search for new and innovative treatments. Advances in immunotherapy and targeted therapy offer hope for patients with this type of cancer.
